# Clinical effect of *rhubarb* on the treatment of chronic renal failure: A meta-analysis

**DOI:** 10.3389/fphar.2023.1108861

**Published:** 2023-04-20

**Authors:** Wei Huang, Yanling Rao, Liang Li, Chengyin Li, Yi An

**Affiliations:** ^1^ Hubei University of Chinese Medicine, Wuhan, China; ^2^ Wuhan Third Hospital, Tongren Hospital of Wuhan University, Wuhan, China; ^3^ The Fifth Hospital of Wuhan, Wuhan, China; ^4^ Hubei Provincial Hospital of Traditional Chinese Medicine, Wuhan, China; ^5^ The Affiliated Hospital of Hubei University of Chinese Medicine, Wuhan, China; ^6^ Hubei Provincial Academy of Traditional Chinese Medicine, Wuhan, China; ^7^ TCM Department, Second Wuhan University of Science and Technology Hospital, Wuhan, China

**Keywords:** *rhubarb*, treatment, chronic renal failure, systematic review, meta-analysis

## Abstract

**Objective:** 1) To evaluate the effificacy of *rhubarb* in the treatment of chronic renal failure (CRF); 2) To explore the safety for rhubarb-based therapy on chronic renal failure.

**Methods:** The randomized and semi randomized controlled trials of *Rhubarb* in the treatment of chronic renal failure in medical electronic databases (up to September 2021) were searched, and meta-analysis was carried out by revman 5.3 software.

**Results:** A total of 2,786 patients were included in 34 literatures, including 1,474 cases in the treatment group and 1,312 cases in the control group. The results of meta-analysis showed that Serum creatinine (SCR) [MD = 123.57, 95% Cl (111.59, 131.96)], Blood urea nitrogen (BUN) [MD = −3.26, 95% Cl (−4.22,−2.31)], Creatinine clearance rate (CCR) [MD = 3.95, 95% Cl (−0.03, 7.93)], Hemoglobin (Hb) [MD = 7.70, 95% Cl (−0.18, 15.58)] and Uric acid (UA) [MD = −42.79, 95% CI (−66.29, −19.29)]. The total effective rate of improving symptoms and signs in chronic renal failure patients [Peto or = 4.14, 95% Cl (3.32, 5.16)].

**Conclusion:** This systematic review and meta-analysis demonstrated that rhubarb has a positive therapeutic effect, which may provide confifidence and some theoretical reference for clinical application to a certain extent. Compared with the control group, *rhubarb* alone or traditional Chinese medicine compound containing *Rhubarb* can significantly reduce Serum creatinine, Blood urea nitrogen and Uric acid, increase Creatinine clearance rate, and improve the total effective rate of symptoms and signs. However, there is no evidence that *rhubarb* is more effective than the control group in increasing hemoglobin. In addition, due to the low quality of research methodology in the included literature, it is necessary to further study high-quality literature to evaluate its efficacy and safety.

**Systematic Review Registration:** https://inplasy.com/inplasy-2021-10-0052/, identifier INPLASY2021100052.

## 1 Introduction

Chronic renal failure (CRF) is a common outcome of various chronic kidney diseases (Padmanabhan et al., 2017). It is a clinical syndrome (Elliott et al., 2003; Plantinga et al., 2009). Statistics from the National Institutes of Health show that chronic renal failure accounts for 7% of the number of people with the disease in the United States, yet accounts for 24% of total healthcare costs (Schwedt et al., 2010). An epidemiological survey of chronic kidney disease in China shows that the prevalence of chronic kidney disease in Beijing up to 9.4% (Sui et al., 2020) (Zhang et al., 2018). It was reported that the number of people suffering from kidney diseases is increasing every year, and the proportion of the total number of people in the world is about 8.33% and the incidence is increasing every year (Williams and Heller, 2016). At present, there are more than 2 million chronic renal failure patients worldwide who rely on dialysis to maintain their lives, and with advances in medical care, their 5-year survival rate has exceeded 80% (Williams and Heller, 2016).

Chronic renal failure is the end stage of many kidney diseases (Bonfante et al., 2001). The urea nitrogen and creatinine excretion through the kidneys is significantly reduced, while the excretion through the intestine is significantly increased in chronic renal failure (Xue et al., 2019). The goal of treatment for chronic renal failure is to delay the progression of renal function and effectively preventing the occurrence of related complications, helping patients to achieve an optimal physiological, biochemical, and psychological state. The main treatment options for chronic renal failure are internal medicine, dialysis (including haemodialysis and peritoneal dialysis) and kidney transplantation (Pawlak et al., 2005). The best treatment options for patients with end-stage renal failure are dialysis and kidney transplantation, but as these two therapies are expensive and have limited sources of donor kidneys, they may be unaffordable and unacceptable to most patients. Therefore, early, and mid-stage conservative medical treatment to mitigation the progression of chronic renal failure is generally accepted.

*Rhubarb*, also known as Da Huang, According to Chinese Pharmacopoeia (Commission, 2015), *rhubarb,* belong to the family Polygonaceae, is derived from the dried root and rhizome of *Rheum palmatum L.*, *Rheum offcinale Baill.*, and *Rheum tanguticum* (*Maxim. ex Regel*) *Balf*., (The Angiosperm Phylogeny Group et al., 2016). *Rhubarb* has been applied in the treatment of CRF in recent years with remarkable clinical efficacy (Zeng et al., 2021). Previous studies have suggested that *rhubarb* treats chronic renal failure mainly as a laxative, causing uremic toxins to be excreted from the bowels *via* the intestines (Yokozawa et al., 1986). The main active constituents of *rhubarb* have been effectively identified and with the development of medicinal chemistry and pharmacology (Kim, 2012). Recently, the result has shown that *rhubarb* can protect kidney function and has specific mechanisms other than laxative action. Anthraquinones are the most studied active ingredients in *rhubarb* and are mainly divided into free and bound forms (Feng et al., 2013). The main components are rheinic acid, aloe emodin, physcion and chrysophanol. *Rhubarb* reduces intestinal uptake of amino acids, leading to a decrease in urea, inhibits proteolysis and leads to a decrease in urea synthesis by the liver (Zhang et al., 2018). It increases the frequency of bowel movements and promotes the excretion of creatinine and urea from the urine and faeces; *Rhubarb* regulates the humoral immune system and expels its antioxidant properties also improve the hypoxic state of the kidneys. In addition, *rhubarb* improves amino acid, nitrogen metabolism and lipid metabolism by inhibiting cell proliferation, reducing extracellular matrix (ECM) deposition and inhibiting tumor necrosis factor (TNF) production (Moon et al., 2006). A study found that argirein synthesized by hydrogen bonding of rhein and L-arginine inhibits the activation of NOX4-dependent O_2_^−^ in rat aortic endothelial cells triggered by palmitic acid, thereby inhibiting endothelial IR and improving vascular function. The traditional Chinese medicine *rhubarb* is useful for the early treatment of chronic renal insufficiency, delaying the development of the disease, and prolonging the life of patients with CRF. Our previous study has also demonstrated that rhubarb ameliorates adenine-induced chronic renal failure in mice by regulating gut microbiota dysbiosis (Wang et al., 2022). Through a systematic review and meta-analysis, rhubarb has positive effects on CRF animals, but it is not clear whether rhubarb is the most important component in the treatment of CRF. Then which is the most effective ingredient of rhubarb in treating CRF? Will the combined action of rhubarb components bring different curative effects? The objective of our systematic review and meta-analysis is to explore the effificacy and active constituents of rhubarb for CRF. Our analysis may provide scientifific reference for the clinical application of rhubarb for CRF.

## 2 Method

### 2.1 Databases searching

Databases searched include the China Biology Medicine disc (CBMdisc), China Academic Journal Network Publishing Database (CAJD) China, Wanfang Database, EMBA, MEDLINE, PUBMED, and Cochrane Library.

### 2.2 Manual searching

Retrieve relevant dissertations and conference papers. When the required information cannot be fully obtained from the retrieved literature report, contact the author of the study to obtain relevant information. At the same time, relevant Chinese journals such as Chinese Journal of kidney disease investigation, Chinese Journal of integrated traditional Chinese and Western medicine, Journal of Traditional Chinese Medicine, Journal of clinical kidney disease, Chinese Journal of Nephrology Dialysis and Transplant, Journal of Traditional Chinese Medicine and other relevant Chinese journals were manually searched to minimize missed detection. The search deadline is September 2021. Key words: chronic renal failure, chronic renal insufficiency, *Rhubarb*, rhein, emodin.

### 2.3 Study population

Diagnostic criteria: according to the criteria formulated at the meeting, the staging criteria are divided into five stages according to the renal function staging criteria proposed in the clinical practice guide for chronic kidney disease (K/DOQI) formulated by the American kidney disease foundation. The treatment group was treated with *Rhubarb* single prescription or traditional Chinese medicine compound containing *Rhubarb* (including traditional Chinese medicine pill, traditional Chinese medicine glue coating, traditional Chinese medicine granule and traditional Chinese medicine decoction) orally, with unlimited dosage form/dose mode. The treatment and follow-up time were more than 2 months.

Exclusion coincidentally: 1): use of Chinese patent medicines, compound Chinese patent medicines or Chinese herbal medicines containing *Rhubarb* ingredients in the control group; 2); use of Chinese herbal enemas containing *Rhubarb* in the treatment group and use of Chinese medicinal preparations of unknown ingredients in the treatment and/or control groups; 3); no control group, or poorly designed, or inappropriate statistical methods, or duplicate publications; 4); failure to meet diagnostic criteria or no diagnostic criteria; 5); observation of staged efficacy; 6); literature for which outcome indicators were not available. 7). When reversible factors that exacerbate renal impairment, such as poor blood pressure control, are present in the presence of renal insufficiency but are not addressed; 8); Dialysis therapy.

### 2.4 Observation indicators

1) Efficacy indicators: BUN, Ccr, Hb. 2). Other observation indicators: number of deaths during treatment and follow-up; number of cases entering renal replacement therapy (dialysis or renal transplantation) during treatment and follow-up; total effective rate; improvement of clinical symptoms and signs, and adverse reactions.

### 2.5 Data extraction and quality evaluation

Two researchers independently extracted the data from the literature that met the requirements, cross-checked the data and discussed with other researchers if they encountered any disagreements. The information extracted included general information about the subjects, the intervention, and outcome indicators. The quality of the literature was evaluated using the Jadad rating scale. The four areas under were evaluated separately: 1): The randomisation method; 2); Allocation concealment and methodological correctness; 3); whether blinding was used; and 4) mention of missing visits or withdrawals, and intentional analysis.

### 2.6 Risk of bias assessment

The methodological quality of the included literature was evaluated by using the “bias risk assessment” tool in Cochrane evaluation manual hand book 5.1.0: 1): random allocation method; 2); allocation concealment scheme; 3); Whether the blind method is used for the research object and the implementer of the treatment plan; 4); Whether the outcome indicators were evaluated by blind method; 5); the result data are completely reported (whether the number of lost visits and withdrawals are described, and whether an intention analysis is conducted); 6); selective reporting of research results; 7); Other sources of bias. Each document is evaluated according to the above points: it is divided into three levels: “yes” stands for low bias, “no” stands for high bias, “unclear” stands for lack of relevant information or uncertainty of bias, which shall be cross checked by two researchers. If there are differences or difficult conditions to be determined, it shall be solved after discussion with other personnel.

### 2.7 Statistical analysis

Statistical analyses were performed using the Revman 5.3 software provided by the Cochrane Collaboration. For notational information, the Peto-Ratio (PetoOR), with its 95% confidence interval (95% CI), and OR (oddsratio), the ratio or dominance ratio, were used, with OR = 1 indicating no difference between the comparison groups. When the subject under study is an adverse event, a OR < 1 represents a possible reduction in the risk of outcome and *vice versa*. For continuous variables with measured information, the weighted mean difference (MD) and its 95% CI were used as the efficacy statistic; when the units of measurement differed, the standard mean difference (SMD) and its 95% CI were chosen as the efficacy statistic, with *p* < 0.05 being a statistically significant difference.

The result of Meta-analysis were presented using Forest plots, and the χ^2^ test and I^2^ test were used for heterogeneity between studies. When *p* > 0.1, I^2^ ≤ 50%, it indicates that the heterogeneity between studies is small or there is no heterogeneity between groups, and Meta-analysis is conducted using the fixed-effects model; when *p* ≤ 0.1, I^2^ > 50%, it indicates that there is a large statistical heterogeneity between studies, and sensitivity analysis is conducted to reduce heterogeneity as much as possible according to the possible heterogeneity factors. If heterogeneity still existed but there was clinical homogeneity, Meta-analysis was performed using a random effects model. Descriptive analysis was used if there was too much heterogeneity, too little data in the literature sample or if the source of data could not be found. An “inverted funnel plot” was used to assess publication bias.

## 3 Result

### 3.1 Study inclusion

**FIGURE 1:**
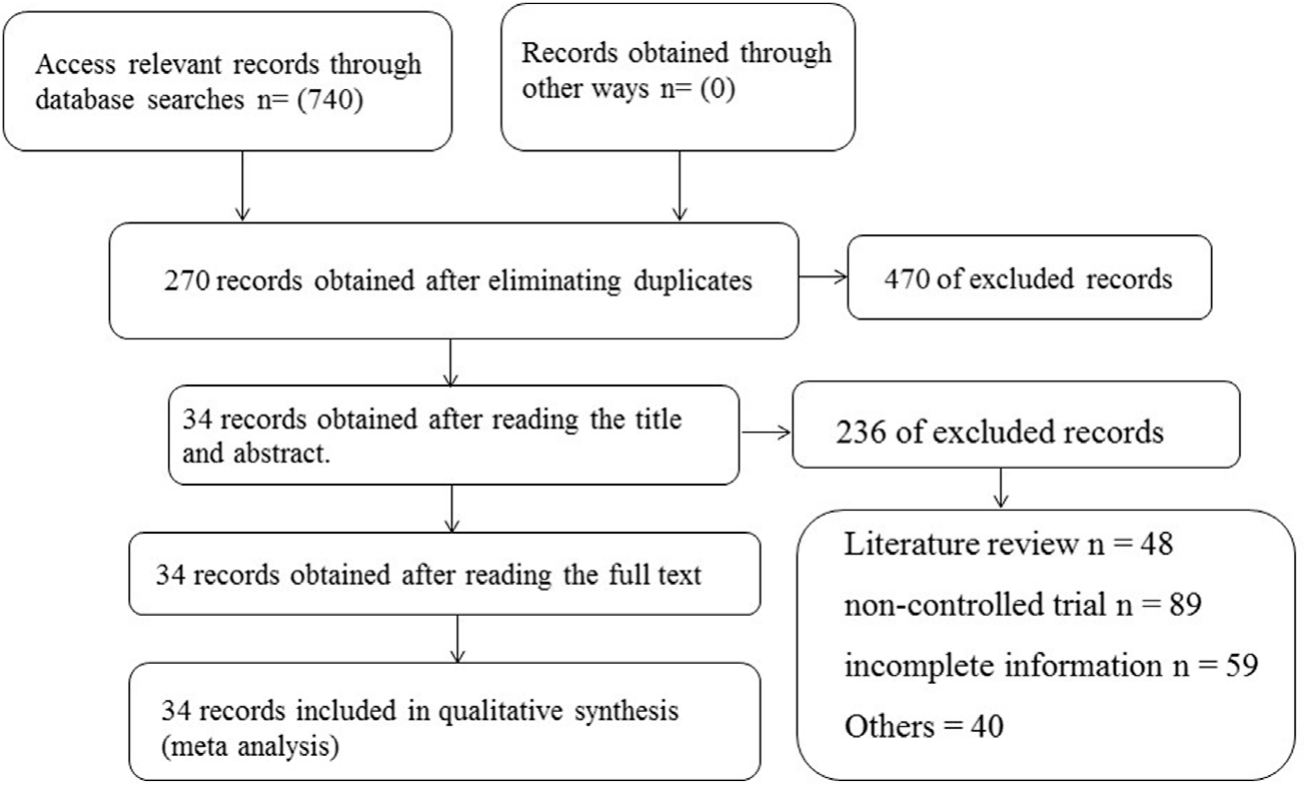
Flow chart of trials selection process for meta-analysis.

The initial search yielded 740 relevant records (145 from CBM, 271 from CNKI, 151 from VIP and 173 from Wanfang). NoteExpress software was used for de-weighting, and the exclusion of ineligible papers was carried out according to the pre-designed de-weighting criteria, and a total of 34 papers were finally identified for inclusion (Yokozawa et al., 1986; Duan, 2005; Gao, 2005; Zhang, 2005; Chang, 2006; Chen and Diao, 2008; Liu and Feng, 2008; Sun, 2008; Yuan, 2008; Cao, 2009; Chen, 2009; Dan, 2009; Rai, 2009; Xue, 2009; Hang, 2010; Jing, 2010; Liu, 2010; Liu and Li, 2012; Ma et al., 2012; Zeng, 2012; Han, 2013; Wang and Sun, 2015; Yan, 2015; Qin, 2016; Williams and Heller, 2016; Li et al., 2017; Zhang et al., 2018; Xue et al., 2019; Yu, 2019; Gong et al., 2020; Jin, 2020; Yang, 2020; Zeng et al., 2021) (Figure 1).

### 3.2 Study characteristics

A total of 34 studies were included, and the total number of patients was 2,786, including 1474 in the treatment group and 1312 in the control group. In addition to 3 studies that did not describe the number of male and female patients, the remaining 31 literatures provided specific data of male and female patients in each group, 1,065 males and 903 females. There were 8 studies which did not describe the original disease. The other 20 studies described the primary diseases, including chronic glomerulonephritis in 810 cases, diabetic nephropathy in 359 cases, Hypertension Nephropathy in 294 cases, chronic nephritis in 253 cases, primary nephrotic syndrome in 32 cases, lupus nephritis in 53 cases, multiple kidneys in 22 cases, and hyperuricemia nephropathy in 18 cases. There were 13 cases of obstructive nephropathy, 12 cases of interstitial nephritis, 4 cases of renal calculi and 61 cases of others. The incidence of primary was chronic glomerulonephritis, diabetic nephropathy and hypertensive nephropathy.

### 3.3 Effectiveness serum creatinine (SCR)

**FIGURE 2:**
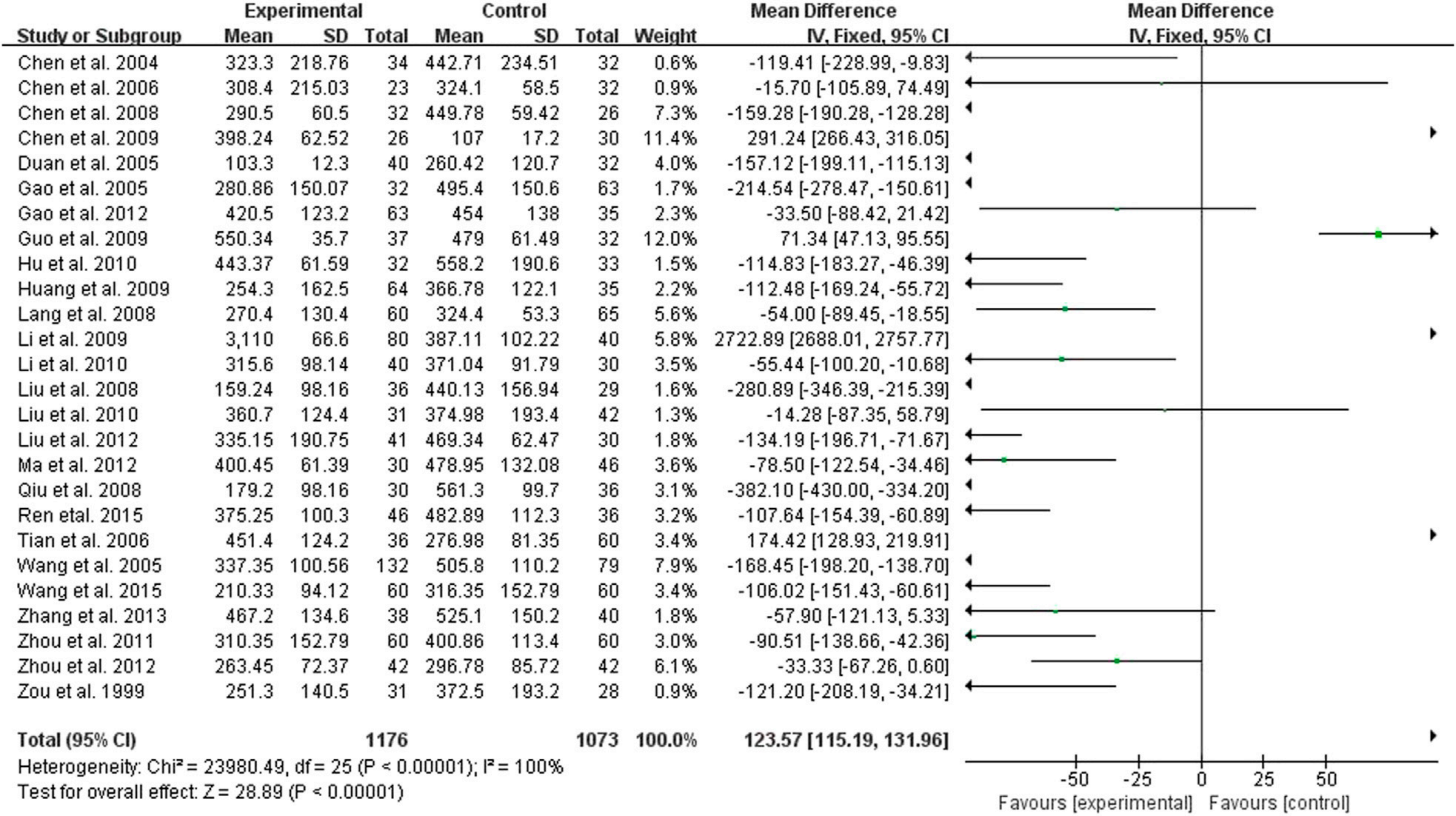
Forest plots showing MD (with 95% CI) for SCR comparing *Rhubarb* with the usual treatment.

Twenty-five studies compared the effect of treatment and control groups on SCR, 1,176 cases in the treatment group and 1,073 cases in the control group, all measured (Figure 2). The test for heterogeneity showed *p* < 0.00001, I^2^ = 100%, and a fixed effects model was used, showing a statistically significant difference (*p* < 0.00001) with MD = 123.57 and 95% Cl of (111.59, 131.96), indicating that in terms of scr reduction, the treatment group had a better effect (Figure 2).

### 3.4 Blood urea nitrogen (BUN)

**FIGURE 3:**
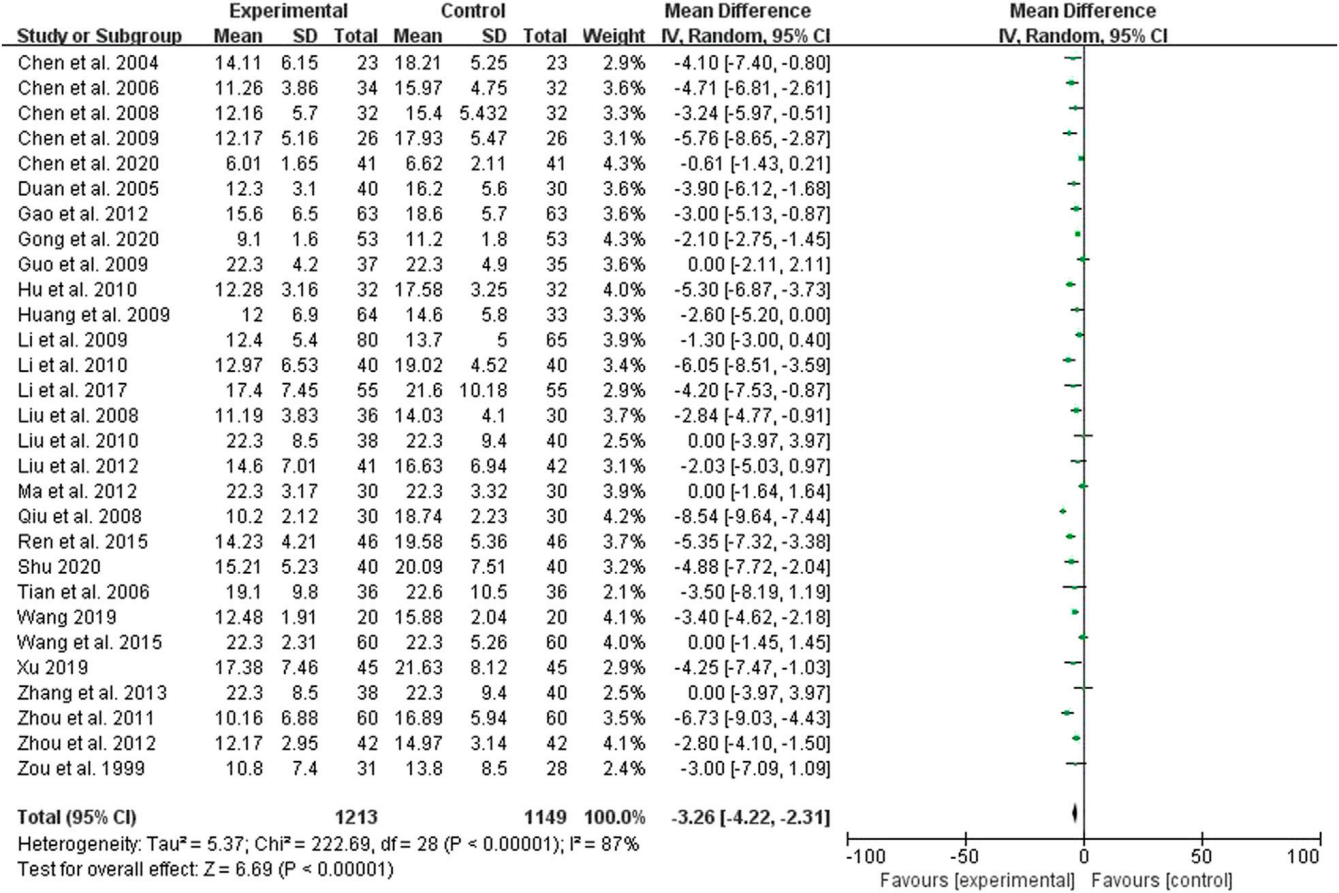
Forest plots showing MD (with 95% CI) for BUN comparing *Rhubarb* with the usual treatment.

Twenty-eight studies compared the effect of the treatment group with the control group on BUN, 1,213 cases in the treatment group and 1,149 cases in the control group, all measured (Supplementary Material; Figure 3). The heterogeneity test showed *p* < 0.00001 and I^2^ = 8%, but there was clinical homogeneity, so a random effects model was used, which showed a statistically significant difference (*p* < 0.00001) with MD = −3.26% and 95% Cl of (−4.22, −2.31), indicating a better treatment effect in reducing BUN in the treatment group compared to the control group (Supplementary Material; Figure 3).

### 3.5 Creatinine clearance rate (CCR)

**FIGURE 4:**
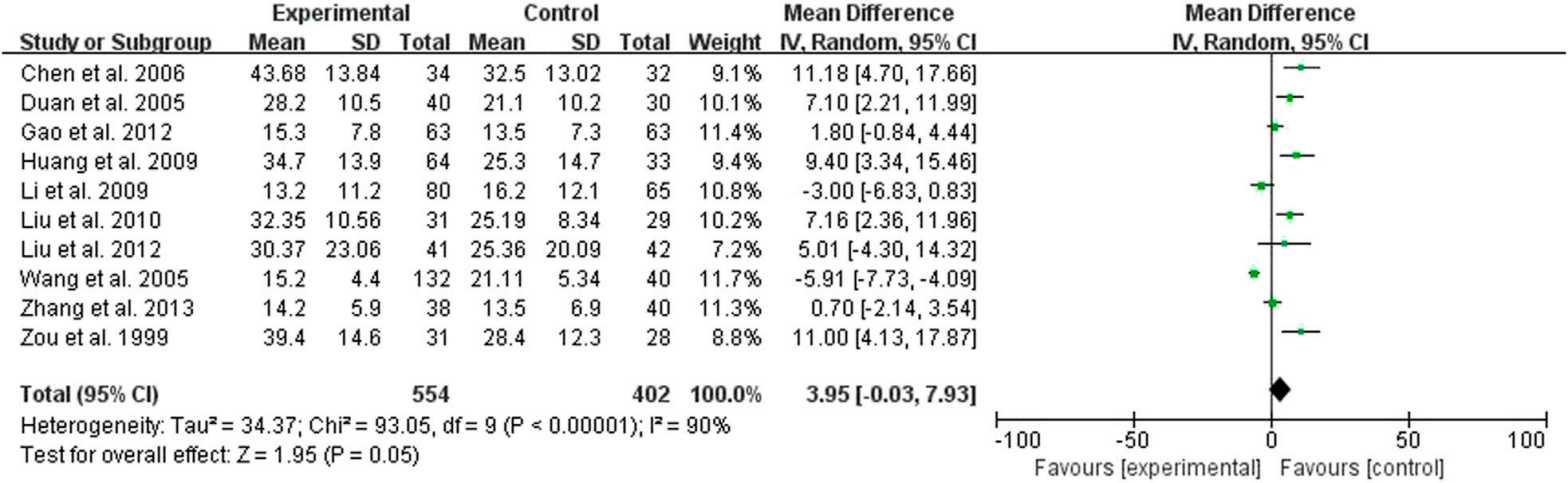
Forest plots showing MD (with 95% CI) for CCR comparing *Rhubarb* with the usual treatment.

Nine studies compared the effect of the treatment group with the control group on CCR, 554 cases in the treatment group and 402 cases in the control group, all measured (Figure 4). The test for heterogeneity showed *p* < 0.00001, I^2^ = 90%, but there was clinical homogeneity therefore using a random effects model, MD = 3.95, 95% Cl (−0.03, 7.93), which was statistically different (*p* = 0.05), indicating that the treatment group was more effective in raising CCR better (Figure 4).

### 3.6 Hemoglobin (Hb)

**FIGURE 5:**
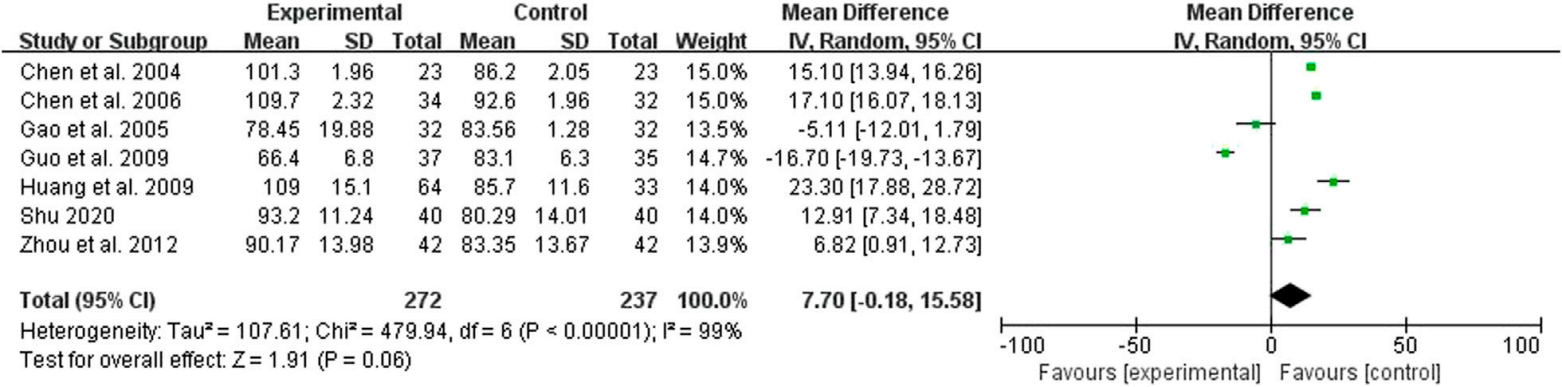
Forest plots showing MD (with 95% CI) for Hb comparing *Rhubarb* with the usual treatment.

Six studies compared the effect of the treatment group with the control group on Hb, 272 cases in the treatment group and 237 cases in the control group, all measured (Figure 5). The heterogeneity test showed *p* < 0.00001 and I^2^ = 99%, but there was clinical homogeneity, so a random effects model was chosen, MD = 7.70 and 95% Cl was (−0.18, 15.58), which was statistically different (*p* = 0.06), indicating the treatment group is not dominant in increasing Hb (Figure 5).

### 3.7 Serum uric acid (UA)

**FIGURE 6:**
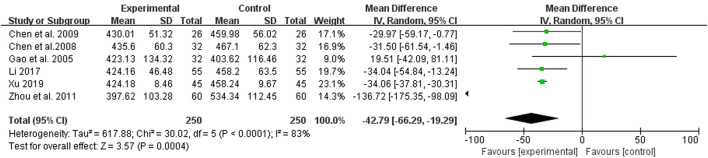
Forest plots showing MD (with 95% CI) for UA comparing *Rhubarb* with the usual treatment.

Five studies compared the effect of the treatment group with the control group on UA, 250 cases in the treatment group and 250 cases in the control group, all measured (Figure 6). The test for heterogeneity showed *p* < 0.00001 and I^2^ = 83%, but there was clinical homogeneity, so a random effects model was chosen with MD = −42.79% and 95% Cl of (−66.29, −19.29), which was statistically different (*p* = 0.0004), indicating a better treatment effect in the treatment group in terms of reducing UA (Figure 6).

### 3.8 The total effective

**FIGURE 7:**
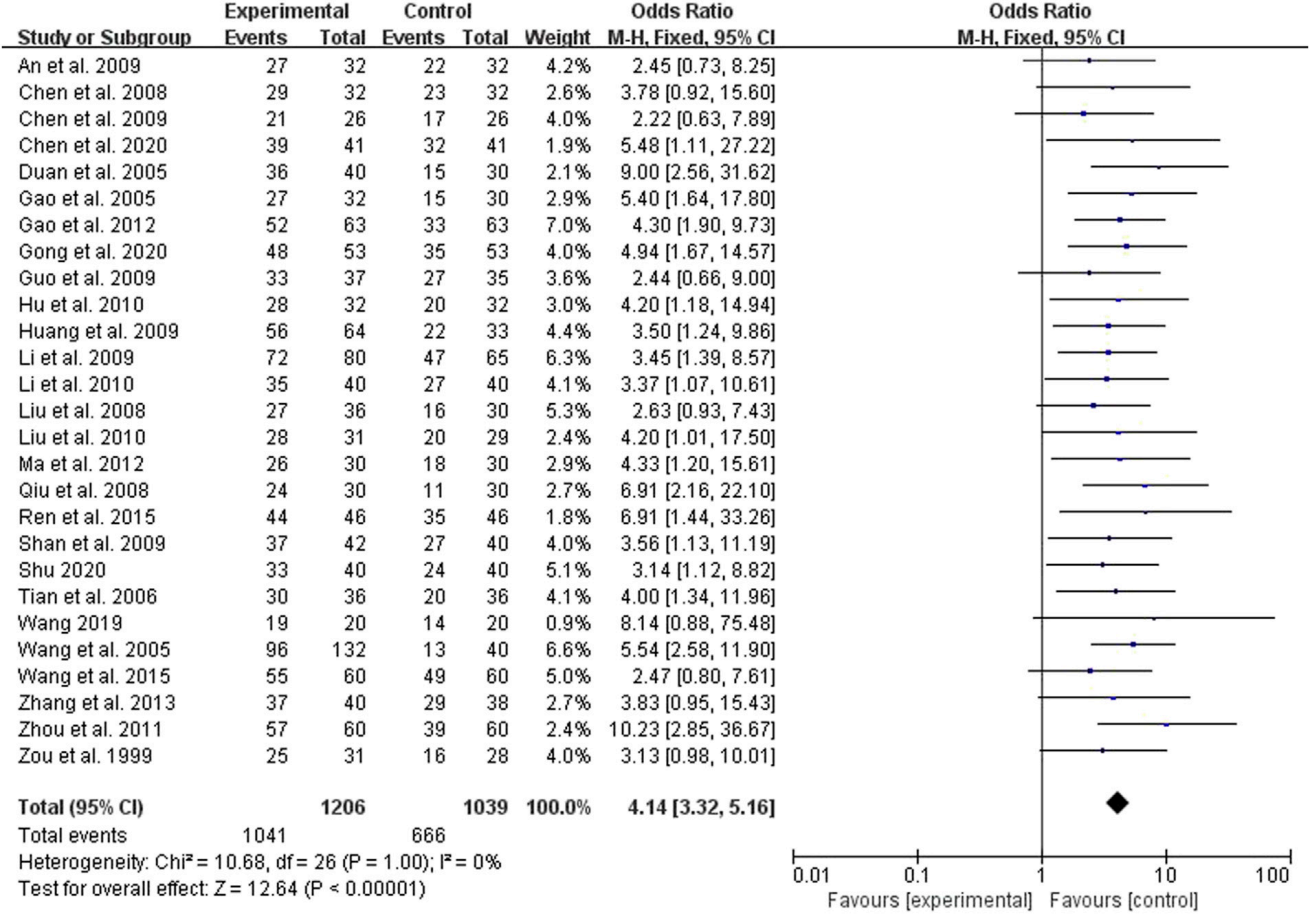
Forest plots showing MD (with 95% CI) for effective comparing *Rhubarb* with the usual treatment.

Twenty six studies compared the effects of the two groups on the total effective rate, 1,041 cases in the treatment group and 666 cases in the control group (Figure 7). The results of heterogeneity test showed that *p* = 1.0, I^2^ = 0%, so the fixed effect model was adopted. The results showed that Peto OR = 4.14, 95% CI (3.32, 5.16), with statistical difference (*p* < 0.00001) (Figure 7). Therefore, the total effective rate of the treatment group was 4.14 times that of the control group, it shows that traditional Chinese medicine enema formula containing *Rhubarb* and basic treatment can improve the total effective rate of symptoms and signs in patients with chronic renal failure.

### 3.9 Incidence of serious adverse events

Eleven studies mentioned adverse reactions, of which 3 articles reported no adverse reactions during the treatment, and 8 studies reported that the adverse reactions were diarrhea, increased number of stools, abdominal pain, and abdominal distension, but they could be improved after adjusting the dosage of *Rhubarb* and symptomatic treatment. The other 23 trials did not mention adverse reactions and other related issues.

### 3.10 Bias estimation

**FIGURE 8:**
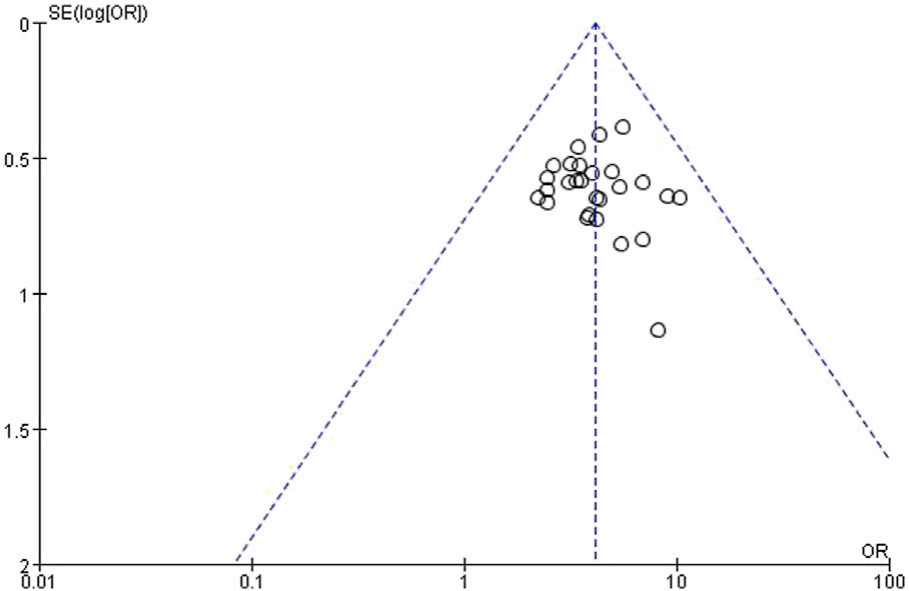
The total efficiency of the funnel plot analysis on *Rhubarb* herbal retention enema treatment of CRF.

The 26 trials involving the comparison of the effects of the treatment group and the control group on the total effective rate were analyzed by “funnel diagram” to understand whether there was publication bias (Figure 8). The results show that the scatter points of the 26 included trials are concentrated on both sides of the straight line and basically symmetrically distributed (Figure 8). It can be considered that the publication bias is small and there is no obvious publication bias.

## 4 Discussion

According to statistics, the number of people with different kidney diseases has increased year by year (Chen et al., 2020). The annual incidence rate of chronic renal failure in natural population is 98–198/100 million, and the incidence rate is increasing year by year (Li et al., 2017). At present, more than 2 million patients with chronic renal failure in the world rely on dialysis to maintain their lives (Gong et al., 2020). With the progress of medical level and the continuous progress of dialysis, renal transplantation and other treatment methods, their 5-year survival rate has exceeded 80%. However, due to the limitation of economic level, only about 10%–15% of patients can receive dialysis therapy, and the high price makes the patients overwhelmed. The early and medium-term treatment of CRF cannot be well solved. At the same time, the government’s cost for renal integration and alternative treatment is also increasing year by year. How to take more measures to reduce the national burden has become an important issue of renal disease concern and research. Therefore, it has become the focus of Nephrology experts to seek effective traditional Chinese medicine from traditional Chinese medicine, which can prevent or delay the progress of renal function damage in CRF patients at an early stage (Li et al., 2017). In this systematic evaluation, the clinical efficacy of *rhubarb* and its compound in the treatment of CRF was comprehensively analyzed and evaluated by comprehensively collecting relevant clinical evidence and using scientific and unified evaluation criteria. Basically, TCM has attracted increasing attention for the potential effective and safe treatment.

From the analysis of results, most of the literatures included in the systematic evaluation have a simple description of the balance between the two groups before treatment, for example, it only shows that the two groups are comparable, and a few trials do not describe the baseline before treatment; Most literatures do not explain the random method, but only mention the word “random,” and do not describe the randomized grouping method in detail; Only one trial reported the double-blind method, and all trials did not report the allocation concealment method in detail. Therefore, there may be implementation bias, measurement bias, or selective bias. Therefore, it can be seen that the quality of the literature methods included in the system evaluation is low, and more rigorous scheme design is needed. In addition, most of the included observations on clinical efficacy use intermediate indicators, such as renal function, CCR, Hb, etc., to statistically evaluate the efficacy, while there are few literatures on endpoint indicators (such as mortality, quality of life, incidence of dialysis due to terminal renal failure and other clinically related long-term follow-up indicators), The overall efficacy and long-term efficacy cannot be well judged.

In the systematic evaluation, the included studies involving the comparison of the effects of the treatment group and the control group on the total effective rate were analyzed by “funnel diagram.” The results showed that the scattered points of the included trials were concentrated on both sides of the straight line, basically symmetrically distributed, and close to the middle of the funnel, indicating that the sample content of the included literature was moderate, so the possibility of publication bias was small.

Cochrane systematic evaluation method was used to review the published RCT and CCT of *rhubarb* and its compound in the treatment of CRF at home and abroad, and systematically evaluate the efficacy indicators, including SCR, BUN, CCR, Hb, and UA and other observation indicators, including total effective rate. The results showed that the efficacy of the treatment group was better than that of the control group in reducing SCR, BUN, increasing CCR, reducing UA and improving the total effective rate of clinical symptoms and signs. Moreover, rhubarb has a powerful blood-activating effect. It is a common medicinal for blood stasis syndrome (Gao et al., 2020). Renal fibrosis (RF) is caused by multiple factors such as inflammation, oxidative stress, apoptosis and so on. After kidney damage, a large number of chemokines and inflammatory factors are produced and released, which promote the apoptosis of renal tubular cells. RF is a common pathological change in a variety of renal diseases that develop into end-stage renal disease, and eventually develop into renal failure. Rhubarb can reduce the reabsorption of amino nitrogen in the intestine, reduce the infiltration of inflammatory cells, improve renal tubular function, so as to protect renal function. Rhubarb can increase plasma osmotic pressure, reduce the high viscosity of blood, and improve the ratio of thromboxane to prostaglandin, play a role in vasodilation, improve microcirculation and increase local blood supply. Sun. (2008) reported Rhein is an important extract of rhubarb. Studies have shown that emodin can delay the process of RIF by promoting the expression of bone morphogenetic protein-7 (BMP-7). Rhein mitigated apoptosis of renal tubular cell as well as renal fibrosis in a UUO rodent model. This curative effect is likely mediated *via* suppression of STAT3 phosphorylation. The curative effect of traditional Chinese medicine enema containing *Rhubarb* in the treatment of CRF is better than that of the control group in improving the main syndromes of traditional Chinese medicine (anorexia, evil and vomiting), but it does not have an advantage over the control group in increasing Hb, and may also be related to the lack of literature on the outcome indicators of Hb.

The incidence of adverse events is an important indicator to evaluate the safety of a treatment strategy. The main adverse reactions of *rhubarb* and its compound in the treatment of CRF are abdominal distension, increased stool frequency, abdominal distension or abdominal pain, evil, vomiting and constipation, but most patients can be relieved after symptomatic treatment. In addition, although rhubarb has the side effect of purgative, the results of our analysis showed that rhubarb-based therapy did not exhibit signifificant side effects. This means it has a high safety profifile in clinical use.

A few studies reported that the blood clock rose during the medication, but all returned to normal after symptomatic treatment, and a few reported liver function damage during the treatment, but continued the treatment with the original scheme, and the liver function could return to normal after the treatment with liver protective drugs. Although a few literatures reported the above-mentioned adverse reactions in the treatment of *rhubarb* and its compound, it did not clearly indicate which drug had side effects. Therefore, it can be known that the reports of adverse reactions in the studies included in this system evaluation are not standardized. Therefore, this system evaluation did not compare the incidence of adverse reactions caused by *rhubarb* and its compound. Therefore, there is no definite conclusion to confirm the safety of *Rhubarb* and its compound therapy. The mechanism of the synergistic effect has not been revealed completely that require more investigations.

Although no convincing conclusion can be drawn according to the existing evidence, Rhubarb and its compound adjuvant therapy for CRF have improved the patient’s condition to a certain extent, and due to the low quality of the included study, it is not possible to make a positive evaluation of its efficacy. In order to recommend the clinical routine application for the treatment of CRF, high-quality large sample, prospective Multi medium and randomized double-blind controlled trials were conducted to extend the observation time, establish endpoint observation, standardize the reporting of adverse reactions and pay attention to the reporting of negative results, so as to clarify the exact efficacy of Rhubarb and its compound in the treatment of CRF. But their value also deserves more exploration in the future.

## 5 Conclusion

In conclusion, this systematic review and meta-analysis demonstrated the positive therapeutic effects of *rhubarb* on patients with chronic renal failure. *Rhubarb* reduced SCR, BUN and UA, increased CCR and improved the total effective rate. However, potential biases remain. Larger, high-quality and rigorously designed studies are needed to obtain more accurate and stable analytical results.

## Data Availability

The original contributions presented in the study are included in the article/Supplementary Material, further inquiries can be directed to the corresponding authors.
