# Supplementary material for: Clinical effect of rhubarb on the treatment of chronic renal failure: A meta-analysis
Source: Front Pharmacol. 2023 Apr 20;14:1108861. doi: 10.3389/fphar.2023.1108861 (PMC10157189; doi:10.3389/fphar.2023.1108861)
Supplement: Supplementary file 1 [file Table1.DOCX]

**SUPPLEMENTARY TABLE** **1** Ingredients and preparation quality control of traditional Chinese medicine compound containing rhubarb therapy in included studies.

| **Study ID** | **TCM formulations** | **Species, concentration（Components）** | **Preparations** | **Quality control** | **Chemical analysis reported?(Y/N)** |
| --- | --- | --- | --- | --- | --- |
| Zhang, 2018 | Xiezuo Decoction | Radix et Rhizoma Rhei,10g | Decoction | Prepared according to Chinese pharmacopeia | Based on previous HPLC research |
| Williams,2016 | Xiezuo Decoction | Radix et Rhizoma Rhei,15g | Decoction | Prepared according to Chinese pharmacopeia | Based on previous HPLC research |
| Xue,2019 | Xiezuo Decoction | Radix et Rhizoma Rhei,10g | Decoction | Prepared according to Chinese pharmacopeia | Based on previous HPLC research |
| Zeng, 2021 | Xiezuo Decoction | Radix et Rhizoma Rhei,12g | Decoction | Prepared according to Chinese pharmacopeia | Based on previous HPLC research |
| Yokozawa, 1986 | Xiezuo Decoction | Radix et Rhizoma Rhei,20g | Decoction | Prepared according to Chinese pharmacopeia | Based on previous HPLC research |
| DL A, 2009 | Shenshuaixiedu Decoction | Radix et Rhizoma Rhei,15g;  concha ostreae,20g;  Taraxaci herba,30g;  [Smilacis Glabrae Rhixoma](https://old.tcmsp-e.com/tcmspsearch.php?qr=Smilacis Glabrae Rhixoma&qsr=herb_en_name&token=fe8ee3eb0193cf96a48f4a1edb39bd54),30g;  [Forsythiae Fructus](https://old.tcmsp-e.com/tcmspsearch.php?qr=Forsythiae Fructus&qsr=herb_en_name&token=fe8ee3eb0193cf96a48f4a1edb39bd54),30g;  [Herba Patriniae](https://old.tcmsp-e.com/tcmspsearch.php?qr=Herba Patriniae&qsr=herb_en_name&token=fe8ee3eb0193cf96a48f4a1edb39bd54),30g;  [Leonuri Herba](https://old.tcmsp-e.com/tcmspsearch.php?qr=Leonuri Herba&qsr=herb_en_name&token=fe8ee3eb0193cf96a48f4a1edb39bd54),30g;  [Persicae Semen](https://old.tcmsp-e.com/tcmspsearch.php?qr=Persicae Semen&qsr=herb_en_name&token=fe8ee3eb0193cf96a48f4a1edb39bd54),15g;  [Carthami Flos](https://old.tcmsp-e.com/tcmspsearch.php?qr=Carthami Flos&qsr=herb_en_name&token=fe8ee3eb0193cf96a48f4a1edb39bd54),15g;  [Aconiti Lateralis Radix Praeparata](https://old.tcmsp-e.com/tcmspsearch.php?qr=Aconiti Lateralis Radix Praeparata&qsr=herb_en_name&token=fe8ee3eb0193cf96a48f4a1edb39bd54),10g;  Sepium,20g;  [Coptidis Rhizoma](https://old.tcmsp-e.com/tcmspsearch.php?qr=Coptidis Rhizoma&qsr=herb_en_name&token=fe8ee3eb0193cf96a48f4a1edb39bd54),10g | Decoction | Prepared according to Chinese pharmacopeia | Based on previous HPLC research |
| H C, 2006 | Dahuang guanchang Decoction | Radix et Rhizoma Rhei,30g;  [Aconiti Lateralis Radix Praeparata](https://old.tcmsp-e.com/tcmspsearch.php?qr=Aconiti Lateralis Radix Praeparata&qsr=herb_en_name&token=fe8ee3eb0193cf96a48f4a1edb39bd54),10g; fossil fragments,30g;  concha ostreae,30g;  serissa serissoide,30g;  [Achyranthis Bidentatae Radix](https://old.tcmsp-e.com/tcmspsearch.php?qr=Achyranthis Bidentatae Radix&qsr=herb_en_name&token=fe8ee3eb0193cf96a48f4a1edb39bd54),30g; [Chuanxiong Rhizoma](https://old.tcmsp-e.com/tcmspsearch.php?qr=Chuanxiong Rhizoma&qsr=herb_en_name&token=fe8ee3eb0193cf96a48f4a1edb39bd54),15g | Decoction | Prepared according to Chinese pharmacopeia | Based on previous HPLC research |
| Chen., 2009 | Huanghuai Decoction | Angelicae Sinensis Radix,15g;  Poria,15g;  Atractylodis Macrocephalae Rhizoma,15g;  Radix et Rhizoma Rhei,10g;  Sophora japonica,10g;,  centella asiatica,10g | Decoction | Prepared according to Chinese pharmacopeia | Based on previous HPLC research |
| Chen, 2008 | Yiqipaidu Decoction | Radix et Rhizoma Rhei,30g;  [Imperatae Rhizoma](https://old.tcmsp-e.com/tcmspsearch.php?qr=Imperatae Rhizoma&qsr=herb_en_name&token=fe8ee3eb0193cf96a48f4a1edb39bd54),30g;  concha ostreae,30g;  [Aconiti Lateralis Radix Praeparata](https://old.tcmsp-e.com/tcmspsearch.php?qr=Aconiti Lateralis Radix Praeparata&qsr=herb_en_name&token=fe8ee3eb0193cf96a48f4a1edb39bd54),10g | Decoction | Prepared according to Chinese pharmacopeia | Based on previous HPLC research |
| QN D., 2016 | Yishen Decoction | Radix et Rhizoma Rhei,20g;  Dandelion,50g;  [concha ostreae](javascript:;),15g | Decoction | Prepared according to Chinese pharmacopeia | Based on previous HPLC research |
| Duan, 2005 | Buyangpaidu Decoction | Radix Rhei Et Rhizome,5g;  Radix Astragali,30g;  Ginseng,18g;  Rhizoma Atractylodis Macrocephalae,18g;  Radix Rehmanniae Praeparata,15g;  Radix Angelicae Sinensis,15g;  Radix Bupleuri,6g;  Radix Scutellariae,6g;  Cortex Cinnamomi,6g;  Radix et Rhizoma Glycyrrhizae,6g;  Fructus Lycii,24g;  [Fructus Ligustri Lucidi](https://old.tcmsp-e.com/tcmspsearch.php?qr=Fructus Ligustri Lucidi&qsr=herb_en_name&token=e1b0d3438dd82223ea36a1aa1ca161ff),24g;  [Hedyotis Diffusae Herba](https://old.tcmsp-e.com/tcmspsearch.php?qr=Hedyotis Diffusae Herba&qsr=herb_en_name&token=e1b0d3438dd82223ea36a1aa1ca161ff),24g;  [Achyranthis Bidentatae Radix](https://old.tcmsp-e.com/tcmspsearch.php?qr=Achyranthis Bidentatae Radix&qsr=herb_en_name&token=e1b0d3438dd82223ea36a1aa1ca161ff),12g;  Radix et Rhizoma Rhei,30g;  Fossilia Ossis Mastodi,30g;  Concha Ostreae,30g;  Herba Taraxaci,30g;  Herba Leonuri,30g;  Radix et Rhizoma Salviae Miltiorrhizae,30g | Decoction | Prepared according to Chinese pharmacopeia | Based on previous HPLC research |
| ZY G,2012 | Qirexiedu Decoction | Radix et Rhizoma Rhei,10g;  Concha Ostreae,30g;  Radix Aconiti Lateralis Praeparata,15g;  Paeoniae Radix Alba,15g;  Radix et Rhizoma Salviae Miltiorrhizae,12g;  Radix Astragali,30g;  Pericarpium Citri Reticulatae,6g;  Poria,30g;  Rhizoma Atractylodis Macrocephalae,10g | Decoction | Prepared according to Chinese pharmacopeia | Based on previous HPLC research |
| Gao,2005 | Dahuang gongying guizhi Decoction | Radix et Rhizoma Rhei,30g;  Dandelion,20g;  Ramulus Cinnamomi,12g;  Rhizoma Smilacis Glabrae,12g;  Fossilia Ossis Mastodi,20g;  Concha Ostreae,20g | Decoction | Prepared according to Chinese pharmacopeia | Based on previous HPLC research |
| R G,2009 | Dahuanggongying Decoction | Radix et Rhizoma Rhei,10g;  Concha Ostreae,20g;  Radix Aconiti Lateralis Praeparata,10g;  Dandelion,10g;  Flos Carthami,10g | Decoction | Prepared according to Chinese pharmacopeia | Based on previous HPLC research |
| Gong,2020 | Rhubarb and Aconite Decoction | Radix et Rhizoma Rhei,50g;  concha ostreae,30g;  Fructus Aurantii Immaturus,30g;  Asiatica,30g;  Radix Aconiti Lateralis Praeparata,15g | Decoction | Prepared according to Chinese pharmacopeia | Based on previous HPLC research |
| Huang,2009 | Tongfuxiezuo Decoction | Radix et Rhizoma Rhei,40g;  Concha Ostreae,30g;  Dandelion,30g;  Herba serissae japonicae,30g;  Flos sophorae,30g;  Radix Aconiti Lateralis Praeparata,10g;  Flos Carthami,10g;  Ramulus Cinnamomi,10g | Decoction | Prepared according to Chinese pharmacopeia | Based on previous HPLC research |
| Hu,2010 | Qiyipaidu Decoction | Radix et Rhizoma Rhei,30g;  Radix Aucklandiae,30g;  Dandelion,30g;  Radix Salviae,30g;  Flos Carthami,30g;  concha ostreae,50g | Decoction | Prepared according to Chinese pharmacopeia | Based on previous HPLC research |
| Li,2010 | Paidu Decoction | Radix et Rhizoma Rhei,30g;  Radix Aconiti Lateralis Praeparata,30g;  Radix Astragali,30g;  Natrii Sulfas,50g;  concha ostreae,50g;  Dandelion,30g;  Radix Pulsatillae,10g | Decoction | Prepared according to Chinese pharmacopeia | Based on previous HPLC research |
| Li,2017 | Dahuangxiezuo Decoction | Flos sophorae,15g;  Herba Violae,15g;  Radix Sanguisorbae,15g;  Radix et Rhizoma Rhei,15g;  Concha Ostreae,30g;  dandelion30g | Decoction | Prepared according to Chinese pharmacopeia | Based on previous HPLC research |
| Liu,2010 | Dahuangfuzi Decoction | Radix et Rhizoma Rhei,30g;  Radix Aconiti Lateralis Praeparata,30g;  Fossilia Ossis Mastodi,30g;  Concha Ostreae,30g;  Herba serissae japonicae,30g;  Radix Astragali,60g;  Chuanxiong Rhizoma,18g;  Dandelion,30g;  Herba Leonuri,30g;  Ramulus Cinnamomi,30g;  Rhizoma Pinelliae Praeparatum,30g | Decoction | Prepared according to Chinese pharmacopeia | Based on previous HPLC research |
| Liu,2008 | Bushenyiqi Decoction | Radix et Rhizoma Rhei,10g;  Dandelion,30g;  concha ostreae,30g;  Fossilia Ossis Mastodi,30g;  Radix Aconiti Lateralis Praeparata,10g;  Radix Salviae,20g | Decoction | Prepared according to Chinese pharmacopeia | Based on previous HPLC research |
| Liu,2012 | Bengdawan  Decoction | Asiatica,60g;  radix fici simplicissimae,60g;  Radix Salviae,30g;  Herba Leonuri,30g;  Radix et Rhizoma Rhei,30g;  Flos sophorae,30g;  [Hedyotis Diffusae Herba](https://old.tcmsp-e.com/tcmspsearch.php?qr=Hedyotis Diffusae Herba&qsr=herb_en_name&token=e1b0d3438dd82223ea36a1aa1ca161ff),30g;  Concha Ostreae,40g;  Radix Sophorae Flavescentis,15g | Decoction | Prepared according to Chinese pharmacopeia | Based on previous HPLC research |
| Ma,2012 | Dahuangjiangzuo Decoction | Radix et Rhizoma Rhei,20g;  Radix Salviae,30g;  Concha Ostreae,50g;  Dandelion,30g;  Flos sophorae,30g;  Semen Persicae,10g | Decoction | Prepared according to Chinese pharmacopeia | Based on previous HPLC research |
| Qiu,2008 | Bushenyangyin Decoction | Radix et Rhizoma Rhei,30g;  Dandelion,30g;  Concha Ostreae,30g;  Rhizoma Smilacis Glabrae,30g;  Rhizoma Pinelliae,10g;  Flos sophorae,10g | Decoction | Prepared according to Chinese pharmacopeia | Based on previous HPLC research |
| Ren,2015 | Qingrejiedu Decoction | Radix et Rhizoma Rhei,30g;  Dandelion,30g;  concha ostreae,30g | Decoction | Prepared according to Chinese pharmacopeia | Based on previous HPLC research |
| Shan,2009 | Xiezuo Decoction | Radix et Rhizoma Rhei,10g;  Concha Ostreae,30g;  Radix Astragali,30g;  Dandelion,30g;  Radix Salviae,20g | Decoction | Prepared according to Chinese pharmacopeia | Based on previous HPLC research |
| Su,2020 | Shenqi Decoction | Concha Ostreae,30g;  Dandelion,30g;  Rhizoma Smilacis Glabrae,30g;  Rhizoma Imperatae,30g;  Radix Astragali,30g;  Radix Salviae,15g;  Lycium Barbarum,15g;  Folium Perillae,15g;  Radix et Rhizoma Rhei,8g;  Radix et Rhizoma Glycyrrhizae,10g | Decoction | Prepared according to Chinese pharmacopeia | Based on previous HPLC research |
| Wang,2015 | Xiezuo Decoction | Radix et Rhizoma Rhei,20g;  Concha Ostreae,30g;  Radix Astragali,40g;  Radix Salviae,30g;  Dandelion,30g | Decoction | Prepared according to Chinese pharmacopeia | Based on previous HPLC research |
| Wang,2005 | Dahuang Decoction | Radix et Rhizoma Rhei,30g;  Concha Ostreae,30g;  Dandelion,30g | Decoction | Prepared according to Chinese pharmacopeia | Based on previous HPLC research |
| Wang,2019 | Rhubarb and Aconite Decoction | Concha Ostreae,18g;  Radix Astragali,18g;  Herba Leonuri,18g;  Radix Paeoniae Rubra,18g;  Radix Aconiti Lateralis Praeparata,10g;  Radix Salviae,10g;  Radix et Rhizoma Rhei,10g;  Ramulus Cinnamomi,6g;  Radix et Rhizoma Glycyrrhizae,6g | Decoction | Prepared according to Chinese pharmacopeia | Based on previous HPLC research |
| Y X,2020 | Dahuang Decoction | Radix et Rhizoma Rhei,15g | Decoction | Prepared according to Chinese pharmacopeia | Based on previous HPLC research |
| Yuan,2008 | Hushenxiezuo Decoction | Radix et Rhizoma Rhei,15g;  Radix Scutellariae,15g;  Concha Ostreae,30g;  Radix Astragali,30g;  Radix Salviae,30g;  Rhizoma Coptidis,10g;  Codonopsis Radix,20g | Decoction | Prepared according to Chinese pharmacopeia | Based on previous HPLC research |
| H Z,2013 | Dahuang Decoction | Radix et Rhizoma Rhei,10g | Decoction | Prepared according to Chinese pharmacopeia | Based on previous HPLC research |
| Chen,2020 | Qihao Decoction | Rhizoma Dioscoreae,10g;  Poria15g,Rhizoma Alismatis,10g;  Radix Rehmanniae Praeparata,10g;  Fructus Corni,10g;  Semen Cuscutae,10g;  Cortex Eucommiae,10g;  Radix Achyranthis Bidentatae,10g;  Caulis Bambusae in Taenia,10g;  Radix et Rhizoma Rhei,10g | Decoction | Prepared according to Chinese pharmacopeia | Based on previous HPLC research |
