# Supplementary material for: Clinical effect of rhubarb on the treatment of chronic renal failure: A meta-analysis
Source: Front Pharmacol. 2023 Apr 20;14:1108861. doi: 10.3389/fphar.2023.1108861 (PMC10157189; doi:10.3389/fphar.2023.1108861)
Supplement: Supplementary file 2 [file Table2.DOCX]

**SUPPLEMENTARY TABLE** **2** Most commonly used ingredients in Rhubarb therapy.

| **Chinese name** | **Pharmaceutical name** | **Species** | **Family** | **N/34(%)** |
| --- | --- | --- | --- | --- |
| Dahuang | Radix Rhei Et Rhizome | Rheum officinaleBaill | Polygonaceae | 34(100%) |
| Pugongying | Dandelion | Taraxacum | Asteraceae Bercht | 19(56%) |
| Dangshen | Codonopsis Radix | Codonopsis pilosula (Franch.) Nannf. | Campanulaceae | 11(32%) |
| Fuzi | Radix Aconiti Lateralis Praeparata | Cynorkis aconitiflora | Ranunculaceae | 11(32%) |
| Huaihua | Flos sophorae | Sophoreae | Fabaceae | 6(18%) |
